# 

**Published:** 1882-05-20

**Authors:** 


### TABLE OF CONTENTS.

ORIGINAL AND SELECTED ARTICLES.

Amputation of the lower extremities for Gangrene from Frost-bite, by Thomas F. Houston, M.D., of Clarksville, Ga., 161. Treatment of Malarial Coma, by Arch. Dixon, M.D., of Kentucky, 165. Report on Vaccine Farms, by James Law, M.D., 168. Puerperal Eclampsia by L. S. Manning, M.D., of Manchester, Ky., 171. The Legal Responsibility of Physicians, 176.

ABSTRACTS AND GLEANINGS.

The Treatment of Syphilis without Mercury—a new Abortive Method, 178. Fractures of the Patella, 179. Treatment of Pneumonia; Blood Enemata, 180. Delirium Tremens, 181. Ozone as a Sleep-producing Agent; Deaths from Chloroform, 182. Do Southern Hogs have Trichinæ; Sulphur for Pimples on the Face; Earache, 183. To Remove Foreign Bodies on the Conjunctiva Beneath Upper Lid, 184. Ergot and Opium in Obstetrics; A prize of $1,000 in Waiting, 185. Treatment of Infantile Diarrhœa, 186. Sudden Death During Forced Depression of Tongue; A Novel Suit Against a Doctor, 187. Tetanus Cured; Permanence of the Scarlatinous Virus, 188. Some of the Consequences of Phimosis and Adherent Prepuce; Paracentesis of the Bladder through the Perineum and Prostate, 189. Bad Effects of Quinine; Almost a Snake Story; the Physician in Court, 190.

SCIENTIFIC ITEMS.

The Hygienic Value of the Electric Light; A Naval Experiment with the Electric Light; Fecundity of Elephants in Confinement, 191. Cotton Fibre; A Tale; The Process; The New York Fire Commissioners, 192.

PRACTICAL NOTES AND FORMULÆ.

Ergot for Lead Poisoning; To Prevent Pitting after Small-pox; Iodine in Typhoid Fever; Diarrhœa in Typhoid, 193. Scrotal Pruritus; Colicky Babies; Grease Eradicator; Dr. Hamilton's Prescription for Epilepsy, 194.

EDITORIALS AND MISCELLANEOUS.

Editorial Notices, 195. Georgia Medical Association, 196. Pamphlets and Papers Received, 198 Book Notices, 199. Recefpted; Special Notices, 200.

